# Supplementary material for: Gastrointestinal Mast Cell Tumor in an African Dormouse (Graphiurus sp.)
Source: Vet Sci. 2022 Sep 11;9(9):497. doi: 10.3390/vetsci9090497 (PMC9504026; doi:10.3390/vetsci9090497)
Supplement: Supplementary file 1 [file vetsci-09-00497-s001.zip › vetsci-1886596-supplementary.pdf]

Supplementary Figure & Legends

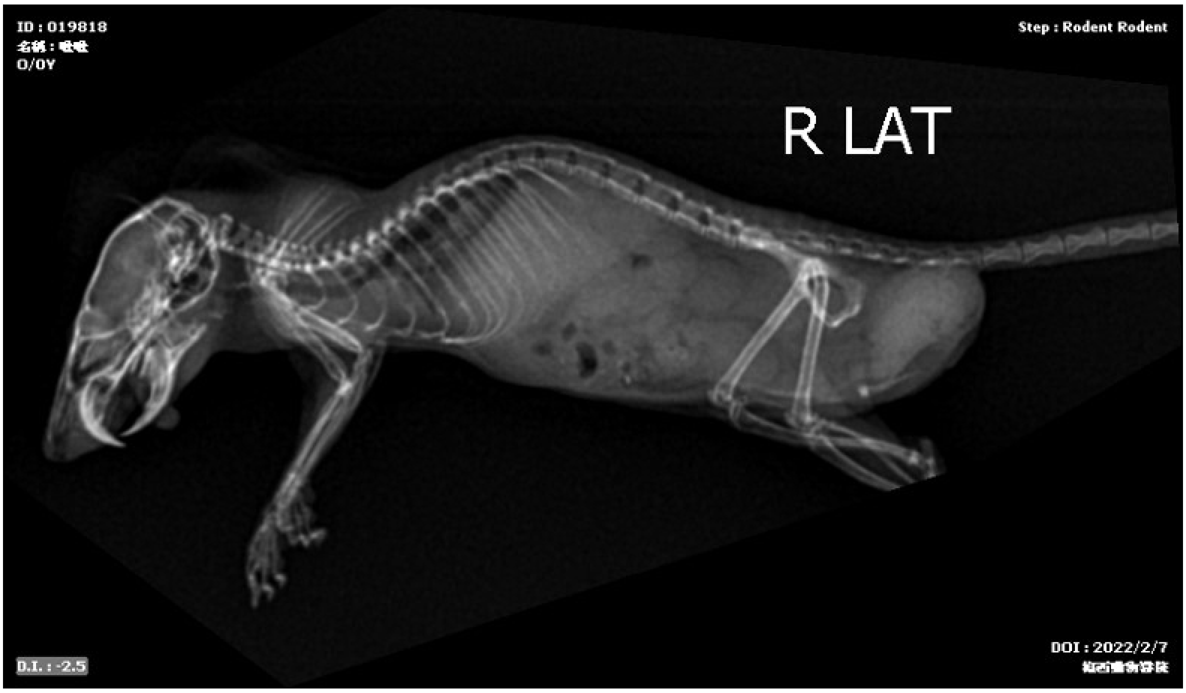

Supplementary Figure S1. Full radiography of the African dormouse. Note the increased opacity of the lungs and distension of intestines.
